# Supplementary material for: A fault-intrusion-tolerant system and deadline-aware algorithm for scheduling scientific workflow in the cloud
Source: PeerJ Comput Sci. 2021 Nov 2;7:e747. doi: 10.7717/peerj-cs.747 (PMC8576554; doi:10.7717/peerj-cs.747)
Supplement: Supplemental Information 6 [file peerj-cs-07-747-s006.zip › Myproject1.1/docs/org/cloudbus/cloudsim/network/datacenter/class-use/Switch.html]

Uses of Class org.cloudbus.cloudsim.network.datacenter.Switch


JavaScript is disabled on your browser.


- Overview
- Package
- Class
- Use
- Tree
- Deprecated
- Index
- Help

- Prev
- Next

- Frames
- No Frames

- All Classes

## Uses of Class org.cloudbus.cloudsim.network.datacenter.Switch

- Packages that use Switch

  | Package | Description |
  | --- | --- |
  |  |  |
  | --- | --- |
  | org.cloudbus.cloudsim.network.datacenter |  |
- - ### Uses of Switch in org.cloudbus.cloudsim.network.datacenter

    Subclasses of Switch in org.cloudbus.cloudsim.network.datacenter

    | Modifier and Type | Class and Description |
    | --- | --- |
    |  |  |
    | --- | --- |
    | `class` | `AggregateSwitch` This class allows to simulate aggregate switch for Datacenter network. |
    | `class` | `EdgeSwitch` This class allows to simulate Edge switch for Datacenter network. |
    | `class` | `RootSwitch` This class allows to simulate Root switch which connects Datacenter to external network. |

    Fields in org.cloudbus.cloudsim.network.datacenter declared as Switch

    | Modifier and Type | Field and Description |
    | --- | --- |
    |  |  |
    | --- | --- |
    | `Switch` | NetworkHost.`sw` |

    Fields in org.cloudbus.cloudsim.network.datacenter with type parameters of type Switch

    | Modifier and Type | Field and Description |
    | --- | --- |
    |  |  |
    | --- | --- |
    | `java.util.List<Switch>` | Switch.`downlinkswitches` |
    | `java.util.Map<java.lang.Integer,Switch>` | NetworkDatacenter.`Switchlist` |
    | `java.util.List<Switch>` | Switch.`uplinkswitches` |

    Methods in org.cloudbus.cloudsim.network.datacenter that return types with arguments of type Switch

    | Modifier and Type | Method and Description |
    | --- | --- |
    |  |  |
    | --- | --- |
    | `java.util.Map<java.lang.Integer,Switch>` | NetworkDatacenter.`getEdgeSwitch()` Get list of all EdgeSwitches in the Datacenter network One can design similar functions for other type of switches. |

- Overview
- Package
- Class
- Use
- Tree
- Deprecated
- Index
- Help

- Prev
- Next

- Frames
- No Frames

- All Classes
